# A forward genetic screen identifies modifiers of rocaglate responsiveness

**DOI:** 10.1038/s41598-021-97765-8

**Published:** 2021-09-16

**Authors:** Leo Shen, Lauren Pugsley, Regina Cencic, HanChen Wang, Francis Robert, Sai Kiran Naineni, Ananya Sahni, Geneviève Morin, Wenhan Zhang, Anastasia Nijnik, John A. Porco, David Langlais, Sidong Huang, Jerry Pelletier

**Affiliations:** 1 Department of Biochemistry, McGill University, Montreal, QC H3G 1Y6 Canada; 2 Department of Physiology, McGill University, Montreal, Canada; 3 McGill Research Center on Complex Traits, McGill University, Montreal, Canada; 4 Department of Human Genetics, McGill University, Montreal, Canada; 5 McGill University Genome Center, McGill University, Montreal, Canada; 6 Department of Microbiology and Immunology, McGill University, Montreal, Canada; 7 Department of Chemistry and Center for Molecular Discovery (BU-CMD), Boston University, Boston, MA USA; 8 Rosalind & Morris Goodman Cancer Research Centre, McGill University, Montreal, Canada; 9 Department of Oncology, McGill University, Montreal, Canada; 10 Centre de Recherche en Biologie Structurale (CRBS), McGill University, Montreal, Canada

**Keywords:** Cancer therapy, Target identification, Target validation, Translation, Chemical biology, Drug discovery, Molecular biology

## Abstract

Rocaglates are a class of eukaryotic translation initiation inhibitors that are being explored as chemotherapeutic agents. They function by targeting eukaryotic initiation factor (eIF) 4A, an RNA helicase critical for recruitment of the 40S ribosome (and associated factors) to mRNA templates. Rocaglates perturb eIF4A activity by imparting a gain-of-function activity to eIF4A and mediating clamping to RNA. To appreciate how rocaglates could best be enabled in the clinic, an understanding of resistance mechanisms is important, as this could inform on strategies to bypass such events as well as identify responsive tumor types. Here, we report on the results of a positive selection, ORFeome screen aimed at identifying cDNAs capable of conferring resistance to rocaglates. Two of the most potent modifiers of rocaglate response identified were the transcription factors *FOXP3* and *NR1I3*, both of which have been implicated in *ABCB1* regulation—the gene encoding P-glycoprotein (Pgp). Pgp has previously been implicated in conferring resistance to silvestrol, a naturally occurring rocaglate, and we show here that this extends to additional synthetic rocaglate derivatives. In addition, *FOXP3* and *NR1I3* impart a multi-drug resistant phenotype that is reversed upon inhibition of Pgp, suggesting a potential therapeutic combination strategy.

## Introduction

Hyperactivation of, or mutations in, genes encoding signalling proteins of the PI3K/Akt/mTOR and MAPK pathways are present in the vast majority of human cancers^1^. As a consequence, the normal cellular constraints on translation regulation mediated at the ribosome recruitment step of initiation are removed, and dysregulated protein synthesis ensues to support remodeling of the tumor cell proteome^2–4^. This dysregulation promotes increased proliferation, resistance to apoptosis, and enhanced metastatic potential^5^. The ribosome recruitment phase of translation is under purview of the PI3K/Akt/mTOR and MAPK signalling networks and is the rate-limiting step in protein synthesis. Eukaryotic initiation factor (eIF) 4F resides at this nexus and its activity dictates the spectrum of mRNAs that enter into the initiation phase and that are subsequently translated^6^. eIF4F consists of (1) an eIF4E subunit which specifically recognizes cap structures, (2) an eIF4A DEAD-box RNA helicase, and (3) a multi-domain scaffolding protein, eIF4G. There are two eIF4A paralogs implicated in translation, eIF4A1 and eIF4A2, but the former is more abundant and better characterized. Whereas eIF4A1 is essential, eIF4A2 is dispensable for cell viability^7^. Repressing eIF4F activity has emerged as a promising anti-cancer therapeutic avenue that capitalizes on the PI3K/Akt/mTOR- and MAPK-driven addictions that many tumor cells display. Targeting eIF4F is expected to be effective across many cancer types and recalcitrant to resistance mechanisms arising from loss of target expression in cancer cells^8,9^.

**Figure 1:**
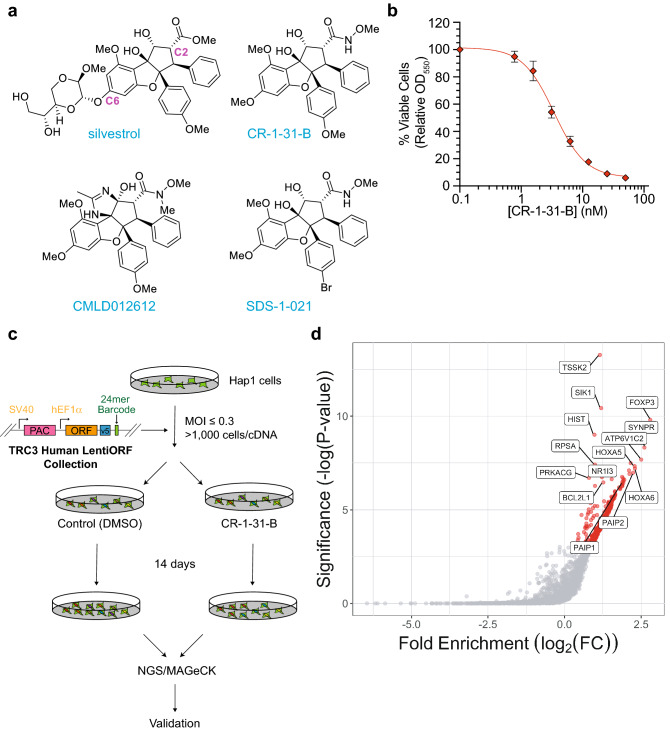
Forward genetic screen for modifiers of CR-1-31-B response. (**a**) Structures of the rocaglates: silvestrol, CR-1-31-B, CMLD012612, and SDS-1-021. (**b**) Titration of CR-1-31-B for 48 h onto Hap1 cells, with cell viability measured by absorbance readings at OD_550_ following SRB staining. n = 3 ± SD. (**c**) Diagram visually depicting the process of cDNA overexpression, positive selection, and pooled functional genomic screening in Hap1 cells. (**d**) Scatter plot depicting the statistical significance (*P*-value as calculated by MAGeCK^61^) of the ORF enrichment plotted against the log_2_ fold change in expression relative to the DMSO control. Labeled genes were selected for further validation.

Several efforts have since explored inhibiting the activities of eIF4F to block ribosome recruitment in cancer, and impeding eIF4A1 function with rocaglates has emerged as a promising avenue^10,11^. Among the options explored, rocaglates (a class of natural products characterized by the densely functionalized cyclopenta[*b*]benzofuran skeleton) exhibit potent activity (Fig. 1a). They impart onto eIF4A and eIF4F gain-of-function properties by inducing clamping between said proteins and RNA to block ribosome recruitment and scanning^12–14^. Furthermore, these compounds exhibit anti-neoplastic activity towards a large range of cancer types in pre-clinical mouse models^5^, and this activity is linked to eIF4A target engagement and inhibition^14–16^. Rocaglates comprise a family of over 200 natural and synthetic compounds with varying potency and bioactivity towards different mRNAs^12^.

To date, we have insufficient knowledge of resistance mechanisms affecting rocaglate responsiveness. It is therefore critical to gain a better understanding of these determinants to inform clinical drug development. At the target level, it has been established that expression of an F163L-mutated eIF4A1 confers rocaglate resistance—as seen in the *Aglaia* genus of plants from which rocaglates were derived^14,16^. Another resistance mechanism has been associated with *ABCB1* (aka P-glycoprotein [Pgp] or multi-drug resistance-1 [MDR1]) levels, where Gupta et al.^17^ showed that inhibition of Pgp by verapamil, or suppression using siRNAs, re-sensitized acute lymphocytic leukemia cells to silvestrol. These results identified *ABCB1*/Pgp as a modifier of the silvestrol response. Here, we report on a genome-wide cDNA overexpression screen undertaken to extend our understanding of determinants of rocaglate sensitivity. Our results identify upstream regulators of *ABCB1* gene expression as additional modifiers of rocaglate sensitivity.

## Results

### A genome-wide functional screen for rocaglate-response modifiers

Chronic myeloid leukemia (CML) is a hematological cancer that is predominantly caused by *BCR-ABL* translocations and for which targeted therapies have revolutionized cancer treatment^18^. Tumors refractory to BCR-ABL tyrosine kinase inhibitors are treated with the translation inhibitor homoharringtonine (HHT, Omacetaxine), which prevents newly formed 80S ribosomes from starting the elongation phase of translation, bringing to a halt all protein synthesis. Despite this profound impact on global translation, a therapeutic index is achievable in the clinic and is thought to result from CMLs being addicted to oncogenic drivers that have short half-lives (e.g. MYC, MCL-1, cyclin D1) which thus become rapidly depleted in transformed cells upon inhibition of translation^19^. More recently, HHT has also been shown to directly bind NF-κB repressing factor (NKRF) and downregulate MYC expression, which may also contribute to HHT’s mechanism of action^20^. Since rocaglates can exert selective effects on the spectrum of mRNAs whose translation are inhibited and are also known to impact translation of critical survival mRNAs^5^, we reasoned that CML cells should also be quite sensitive to rocaglates.

We have previously characterized > 200 rocaglates for their in vitro potency towards inhibiting translation initiation, as well as their RNA sequence requirements for inducing clamping onto RNA^12^. The structures of four key members (silvestrol, CR-1-31-B, CMLD012612, and SDS-1-021) are shown in Fig. 1a. Among these, CR-1-31-B is an especially potent inhibitor of eIF4A and has been an important tool for targeting eIF4A in biological assays. When tested against Hap1 CML cells, CR-1-31-B showed potent activity with an IC_50_ of 3.4 nM (Fig. 1b). The exquisite sensitivity of Hap1 cells to CR-1-31-B offered the opportunity to undertake an unbiased positive selection genetic screen in search of modifiers of CR-1-31-B response. To this end, we chose to perform the screen using 5 nM CR-1-31-B (~ IC_60_) since this concentration was sufficient to dampen protein translation (Fig. S1a). Moreover, cell toxicity at 5 nM was due to eIF4A1 target engagement, as determined by the rocaglate-responsiveness of eHAP1 cells genetically modified to harbor the eIF4A1^F163L^ rocaglate-resistant allele and in which we also ablated the second eIF4A paralog, eIF4A2 (Fig. S1b)^21^. [eHAP1 cells are isogenic to Hap1 cells but have been modified to be truly haploid^22^.] The results indicate that the effects of 5 nM CR-1-31-B on Hap1 cell viability is a consequence of eIF4A1 target engagement.

We then constructed a pooled genome-wide cDNA expression library from the arrayed and sequence-verified MISSION^®^ TRC3 Human LentiORF Collection containing 16,000 ORF clones. This pooled lentiviral library was used to infect Hap1 cells at a multiplicity of infection (MOI) of ≤ 0.3 and a representation of > 1000 cells/cDNA clone (Fig. 1c). Cells were then divided into two identical populations cultured in either DMSO or 5 nM CR-1-31-B for two weeks. Following selection, genomic DNA was isolated from these two populations and the relative abundance of cDNAs determined by Next-Generation Sequencing (NGS) of the unique barcodes present in each vector. This enabled us to identify cDNAs enriched in the CR-1-31-B-treated Hap1 cell population using the MAGeCK statistical software package (Fig. 1d, Table S1).

### Validation of hits from cDNA screening

**Figure 2:**
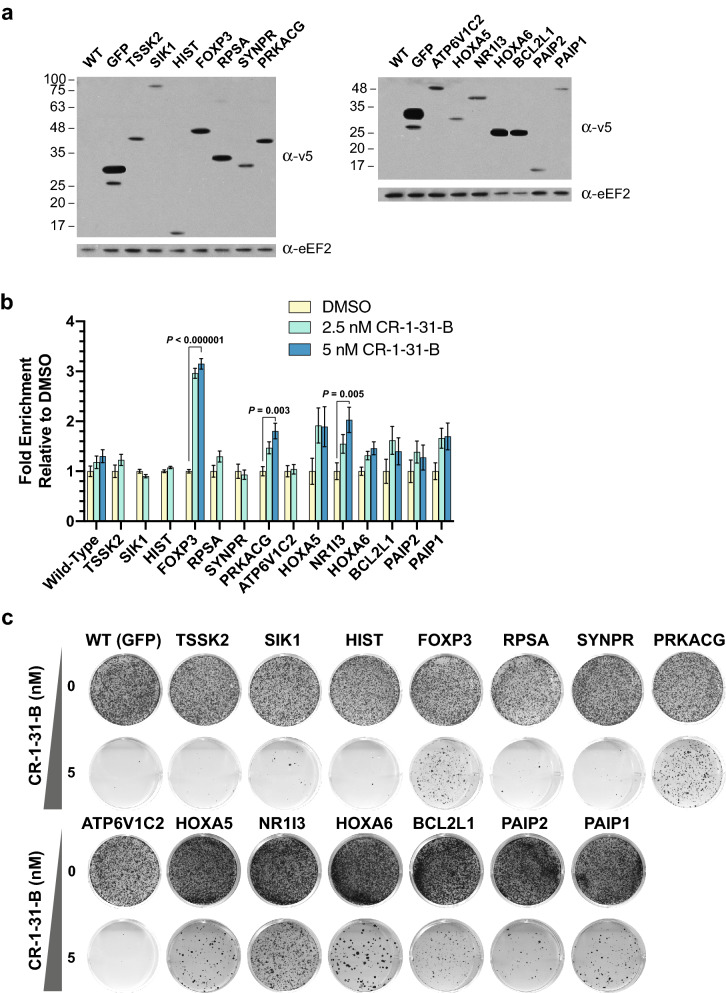
Validation of top candidates for resistance against CR-1-31-B. (**a**) Western blot verifying expression of V5-tagged cDNA constructs transduced into Hap1 cells. Uncropped scans are in Fig. S4. (**b**) Competition assay, wherein negative control, GFP-expressing Hap1 cells were mixed 1:1 with the cell line of interest, and subsequent enrichment of the GFP-negative fraction (cell line of interest) was monitored via flow cytometry following CR-1-31-B treatment (2.5 or 5 nM). Measurements were obtained on day 9 and computed as fold changes over the DMSO-treated condition. For some cell lines, data for 5 nM CR-1-31-B treatment is missing as too few cells survived to reach the event threshold (5000) during acquisition. n = 3 ± SD, statistical significance determined using 2-way ANOVA followed by Dunnett's multiple comparisons test. The other comparisons in this panel do not fall below 0.05 and were not further considered. (**c**) Representative experiments from colony formation assays in various Hap1 cell lines following CR-1-31-B treatment for 6–14 days are shown. One representative experiment from at least three biological replicates is presented.

To validate the results of our screen, we obtained the lentiviral vectors expressing 14 individual cDNAs among the top 25 hits. These were individually transduced into wild-type Hap1 cells, which were then subjected to puromycin selection. A GFP-expressing control was also generated using the same method. All cell lines ectopically expressed recombinant proteins of the expected molecular mass, as assessed by Western blots (Fig. 2a). To determine if transduced cells showed a growth advantage in the presence of CR-1-31-B, they were mixed at a ~ 1:1 ratio with parental Hap1 cells that had been infected with a GFP-expressing lentiviral vector (Fig. 2b). Cells were co-cultured in the presence of DMSO, 2.5 nM CR-1-31-B, or 5 nM CR-1-31-B for 9 days, with flow cytometry readings taken every 3 days to determine the fraction of GFP^−^/GFP^+^ cells (Fig. 2b). Significant enrichment was seen at day 9 for cells expressing FOXP3, PRKACG, and NR1I3 (Fig. 2b)*.* Response to CR-1-31-B was also assessed in a colony formation assay, where resistance (of varying robustness), following a 14-day drug exposure, was obtained with *FOXP3, PRKACG, HOXA5, NR1I3, HOXA6, BCL2L1, PAIP2,* and *PAIP1-*transduced cells (Fig. 2c). In sum, these experiments demonstrate that 3/14 tested cDNAs (*FOXP3, PRKACG, NR1I3*) obtained from the pooled screen indeed conferred resistance in both the competition and colony formation assays, while an additional 5/14 (*HOXA5, HOXA6, BCL2L1, PAIP2, PAIP1*) showed slight to moderate resistance in the colony formation assay. We therefore pursued FOXP3-, PRKACG-, and NR1I3-expressing cells to determine if they could also confer resistance to other eIF4A inhibitors.

To further examine the resistance profiles of *FOXP3-, PRKACG-,* and *NR1I3-*transduced cells, we tested the rocaglates: silvestrol (a well-characterized rocaglate), CMLD012612 (from the newer amidino-rocaglate family^23^), and SDS-1-021 (another potent rocaglate with a structure similar to CR-1-31-B) (Fig. 1a). These were included here to obtain additional information on the biological properties of each compound series. Moreover, pateamine A (Pat A), a natural product that also clamps eIF4A to RNA^12,21,24^, and hippuristanol (Hipp), a third eIF4A inhibitor that functions in a manner distinct from rocaglates and Pat A by binding to the eIF4A C-terminal domain and inhibiting RNA binding^25^, were tested (Fig. 3a). When these cells were tested in competition assay and colony formation assays, we found that ectopic expression of *FOXP3-, PRKACG-,* and *NR1I3* cDNAs provided cross-resistance to silvestrol and CMLD012612 (Fig. 3b,c). In addition, FOXP3- and NR1I3*-*expressing Hap1 cells provided modest cross-resistance to PatA in the competition assay and even more apparent resistance in the colony formation assay (Fig. 3b,c). In contrast, none of the transduced cell lines were resistant to hippuristanol (Hipp) (Fig. 3c). FOXP3- and NR1I3-expressing Hap1 cells also conferred resistance to SDS-1-021 in a colony formation assay (Fig. 3c).

**﻿Figure 3:**
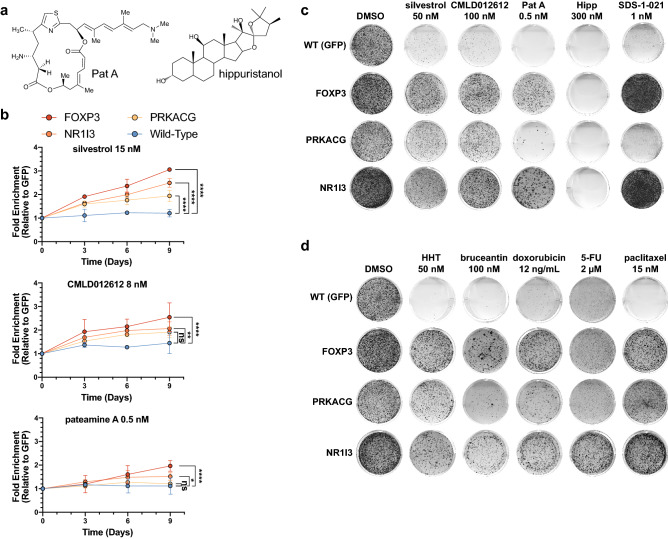
*FOXP3* and *NR1I3* confer a robust multi-drug cross-resistant phenotype. (**a**) Structures of Pat A and Hipp. (**b**) Competition assays with GFP^+^-transduced or gene-of interest-transduced Hap1 cells exposed to silvestrol, CMLD012612, or Pat A. Fold change relative to the GFP^+^ transduced cell line on day 0 is shown. n = 3 ± SD, statistical significance determined using a two-way ANOVA, followed by Dunnett’s multiple comparison test. *P*-value cut-offs: ns (*P* > 0.05), *(0.05 ≥ *P* ≤ 0.01), **(0.01 ≥ *P* ≤ 0.001), ***(0.001 ≥ *P* ≤ 0.0001), **** (*P* ≤ 0.0001). (**c**) Representative experiments from colony formation assays in the indicated Hap1 cell lines following treatment with eIF4A inhibitors for 6–21 days are shown. n ≥ 3. (**d**) Representative experiments from colony formation assays in the indicated Hap1 cell lines following exposure to compounds for 6–21 days. n ≥ 3.

We then further expanded the compound test set to include two inhibitors of translation elongation, the aforementioned homoharringtonine [HHT] as well as bruceantin [Bruc]—compounds that stall newly formed 80S complexes at the initiation codon. Three front-line chemotherapeutics (Doxorubicin [DXR], 5-Fluorouracil [5FU], and paclitaxel [PTX]) were also tested (Fig. 3d). Here again, FOXP3- and NR1I3-expressing Hap1 cells conferred robust resistance, whereas the response from PRKACG-expressing cells was more tempered when exposed to Bruc and 5FU. We note that the cells used in these experiments showed no differences in doubling time, when compared to GFP-transduced or parental Hap1 cells, suggesting a mechanism beyond only higher basal growth rate to be behind the resistance (Fig. S2). Additionally, FOXP3 and NR1I3 protein levels were only elevated in cells transduced with the respective cDNA lentiviral vectors when compared to uninfected, parental cells or GFP-infected control cells, which showed low to undetectable levels (Fig. S3). These results demonstrate that FOXP3 and NR1I3 confer a robust multi-drug resistant (MDR) phenotype when overexpressed in Hap1 cells.

### RNASeq identifies *ABCB1* as a FOXP3-upregulated target

**Figure 4:**
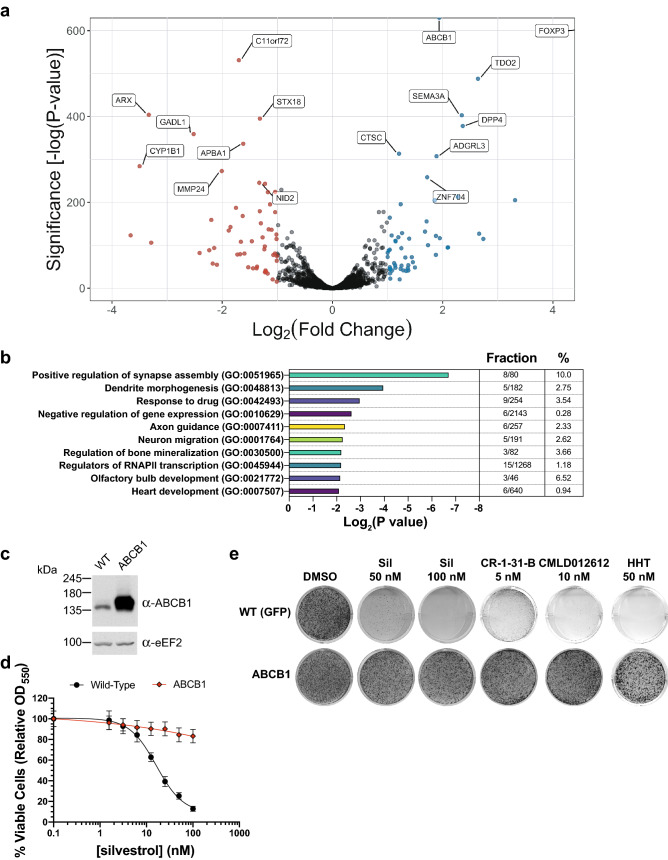
Transcriptomic profiling by RNAseq from FoxP3-expressing cells. (**a**) Volcano plot of RNASeq results from FoxP3-expressing compared to GFP-expressing cells, as determined by edgeR. Blue dots: transcripts with a log_2_FC ≥ 1. Red dots: transcripts with a log_2_FC ≤ − 1. Summary of results (55 genes significantly upregulated, 53 significantly downregulated, *P* < 0.05). (**b**) GO enrichment analysis predictions of the effects of *FOXP3* overexpression on Hap1 cells. Shown are the logarithms of the Benjamini–Hochberg corrected *P*-values. Overlap represents the number of genes per GO term, represented as fractions and percentages. (**c**) Western blot documenting expression of ABCB1 in transduced Hap1 cells. Uncropped scans are in Fig. S5. (**d**) Titration of silvestrol onto the indicated Hap1 cells, with cell viability measured 48 h later by SRB staining. n = 3 ± SD. (**e**) Representative experiments from three colony formation assays in Hap1 cells treated with the indicated compounds for 6–14 days.

FOXP3 and NR1I3 are two, fairly well-studied transcription factors (see “Discussion”). To investigate the molecular mechanism underlying the FOXP3-mediated MDR phenotype, we undertook a transcriptomics approach by performing RNASeq in Hap1 cells ectopically expressing FOXP3 or GFP (control). We found 55 mRNAs that were significantly upregulated with a ≥ twofold change and 53 significantly downregulated mRNAs with a ≤ twofold change (*P* ≤ 0.05) (Fig. 4a, Table S2). Genes significantly upregulated in *FOXP3*-expressing Hap1 cells were enriched for biological processes related to neuronal development, drug response, gene expression regulation, bone mineralization, and olfactory bulb and heart development (Fig. 4b). Notably, we found via gene ontology analysis that FOXP3 overexpression was associated with genes involved in response to drug (Fig. 4). Among this group, *VEGFC*, *LPL*, *CCND1*, *GNAO1*, *BCHE*, *FYN*, *SEMA3C*, *LOX*, and *ABCB1*, were enriched for in FOXP3-expressing cells relative to the GFP-expressing cells. *FOXP3* expression itself served as an internal control and was indeed found to be the highest overexpressed mRNA (Fig. 4a). It struck us that *ABCB1* emerged as the top-ranked gene candidate upregulated by FOXP3 (*P* < 0.0001), given the previously reported ability of ABCB1 to confer MDR to conventional chemotherapeutics as well as to silvestrol^17^. We thus ectopically expressed *ABCB1* in Hap1 cells and found this was indeed sufficient to confer resistance to silvestrol, CR-1-31-B, CMLD012612, and the unrelated translation inhibitor HHT (Fig. 4c–e).

### Inhibitors of ABCB1 reverse the MDR phenotype in FOXP3- and NR1I3-expressing Hap1 cells

**Figure 5:**
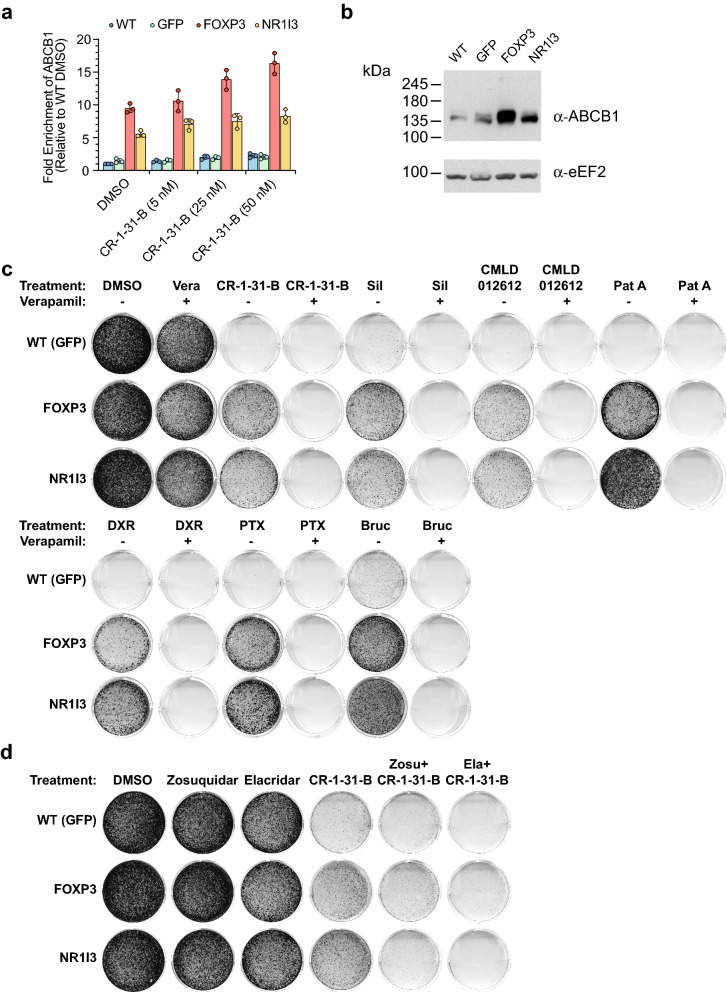
Inhibition of ABCB1 reverses the MDR phenotype in FOXP3- and NR1I3-expressing cells. (**a**) *ABCB1* mRNA levels in wild-type (WT), *GFP*-, *FOXP3*-, and *NR1I3*-expressing Hap1 cells were measured by RT-qPCR across 4 conditions (DMSO, 5/25/50 nM CR-1-31-B for 6 h). n = 3 ± SD. (**b**) Western blot documenting ABCB1 expression in wild-type (WT), GFP-, FOXP3-, and NR1I3-expressing Hap1 cells. Uncropped scans are in Fig. S5. (**c**) Representative experiments from colony formation assays in Hap1 cells following compound treatment in the presence or absence of verapamil for 4–14 days. n ≥ 3. Compounds and concentrations were: 10 µM verapamil (Vera), 5 nM CR-1-31-B, 50 nM silvestrol (Sil), 10 nM CMLD012612 (012612), 1 nM Pat A, 12 ng/mL doxorubicin (DXR), 15 nM paclitaxel (PTX), 10 nM bruceantin (Bruc). (**d**) Representative experiments from colony formation assays in Hap1 cells following 5 nM CR-1-31-B treatment in the presence or absence of zosuquidar or elacridar for 4–14 days. n ≥ 3.

In addition to FOXP3, NR1I3 has also been implicated in the regulation of *ABCB1* (see “Discussion”). Therefore, to probe whether *ABCB1* levels changed upon ectopic expression of FOXP3 and NR1I3, we undertook RT-qPCR analysis to quantitate *ABCB1* mRNA levels in parental Hap1 *GFP*-, *FOXP3*-, and *NR1I3*-transduced cells (Fig. 5a). The results indicate that *ABCB1* mRNA levels were elevated in both *FOXP3*- and *NR1I3*-expressing cells (Fig. 5a). We observed that treatment of these cell lines with CR-1-31-B for 6 h further induced expression of *ABCB1* mRNA levels by ~ 1.5-fold. An increase in ABCB1 at the protein level was also evident by Western blotting (Fig. 5b). To determine the extent to which elevated ABCB1 conferred drug resistance, these isogenic cells were exposed to rocaglates, PatA, doxorubicin, paclitaxel, and bruceantin in the absence or presence of verapamil, a first-generation MDR reversal agent. In all cases tested, verapamil clearly reversed the MDR phenotype associated with FOXP3 and NR1I3 ectopic expression (Fig. 5c). Consistent with this, two additional potent and more specific third-generation ABCB1 modulators, zosuquidar and elacridar, were also able to reverse the CR-1-31-B-resistant phenotype in both *FOXP3*- and *NR1I3*-transduced cells (Fig. 5d).

## Discussion

Knowledge on determinants of resistance mechanisms is critical to inform clinical drug development. Elevated expression of pro-survival proteins (*e.g.* Mcl-1, Bcl-2, Bcl-X_L_) can lead to drug resistance and has been associated with altered sensitivity to rocaglates^26–28^ as well as hippuristanol^29^. We note that cDNAs encoding several pro-survival proteins were indeed significantly enriched in our screen (*BCL2L1*, *BCL2L2*, *MCL1*, *BCL2L10*); however, they ranked lower than the genes we focussed on in this study (Table S1). Moreover, the multidrug efflux transporter, ABCB1*,* has previously been reported to confer resistance to silvestrol^17,29^. ABCB1 is an ATP-binding cassette (ABC) superfamily member alongside MDR1-like protein ABCC1 (MRP1) and ABCG2 (BCRP); taken together, these constitute major drug efflux transporters^30^. Increased levels of ABC transporters is a cause of drug resistance across a large number of tumor types^31^. Two reports have also documented a role for the *KEAP1-NRF2* axis in modulating responsiveness to the rocaglates, silvestrol, CR-1-31-B and eFT226^32,33^. KEAP1, CUL3, and CAND1 are part of the E3 ligase complex responsible for degrading NRF2, and CRISPR-based screens targeting these negative regulators have found that the resulting increase in NRF2 is associated with rocaglate-resistance^32,33^. They postulate that NRF2’s effect in increasing global protein production by maintaining the reduced state of key translational regulators such as eEF2^34^ in conjunction with its ability to increase the expression of eIF4A-dependent oncoproteins such as MYC and BCL2 may be behind the resistance^32,33^.

Forward genetic screens are powerful approaches by which to identify drug response modifiers. Screens taking advantage of cDNA expression libraries are complementary to those using CRISPR-based knock-out approaches. As with any screen, cDNA libraries are not without their shortcomings, however. Single-copy integration may not be sufficient to confer resistance in a positive selection setting; not all cDNA isoforms of genes are present in current libraries; vector-based expression may be sub-optimal for phenotype selection; and skewed clone representation due to variation in packaging efficiencies resulting from differences in cDNA insert size can all affect screening success. These limitations may explain why *ABCB1* itself was not identified as significantly enriched in our original screen (Table S1). We note that although the NRF2 (aka NFE2L2) cDNA was present in our library pool, its ectopic overexpression did not lead to CR-1-31-B resistance, which we postulate is likely due to KEAP1/CUL3/CAND1 E3 ligase’s continued presence and degradation of NRF2. Incorporating a parallel or complementary screen utilizing a CRISPR gene activation approach (CRISPRa), or a knockdown/knockout approach, would likely help overcome some of these shortcomings. Furthermore, overlapping the results of a cDNA and CRISPRa screen could lead to a gene list with a higher validation rate than obtained herein.

Nonetheless, despite these limitations, we were able to identify modifiers of the rocaglate response in Hap1 cells. FOXP3 is a transcription factor predominantly expressed in T cells that plays a critical role in immune regulation^35^. Overexpression of FOXP3 in other settings, such as non-small cell lung cancer (NSCLC), has been linked to elevated cell proliferation, invasion, and increased cell migration in vitro and metastasis in vivo^36^. Consistent with its predicted role as a transcription factor, ectopic expression of FOXP3 in NSCLC leads to suppression of E-cadherin and upregulation of *N*-cadherin, vimentin, snail, slug, and MMP9^36^. Our results extend the role of FOXP3 to chemotherapy response via upregulation of *ABCB1*, which can lead to resistance towards not only rocaglates, but also other inhibitors of translation as well as front-line chemotherapeutics (Fig. 3c,d). Our results with inhibitors of ABCB1 (verapamil, zosuquidar, and elacridar) indicate that the drug resistance obtained upon ectopic expression of *FOXP3* is predominantly mediated through *ABCB1* upregulation (Fig. 5c,d).

NR1I3 is a nuclear receptor that, upon binding various xenobiotics, is known to regulate the expression of drug-metabolizing enzymes and transporters^37^. *ABCB1* is one of several transporters known to be regulated by NR1I3, with others being MRP2, MRP3, and several OATP (organic anion transporting polypeptide) family members^37^. NR1I3 has been shown to bind to an enhancer element 7.8 kbp upstream of the *ABCB1* gene, thereby activating its expression^38^. As with *FOXP3*, our results demonstrate that ectopic expression of *NR1I3* is sufficient to stimulate expression of *ABCB1* in Hap1 cells (Fig. 5a). ABCB1 appears to be the principal driver of resistance towards CR-1-31-B, rather than another transporter in Hap1 cells, as indicated by the results using ABCB1 inhibitors (Fig. 5c,d).

Additional hits that emerged from our screen and that validated in the colony formation assay (Fig. 2) were *PRKACG*, *HOXA5*, *HOXA6*, *PAIP1*, and *PAIP2*. PRKACG is the gamma catalytic subunit of cAMP-dependent protein kinase A (PKA). Cyclic adenosine monophosphate is a critical secondary messenger that has been found to induce drug resistance against several chemotherapeutics in APL and CML cancers^39–41^. PKA activation has also been shown to promote cancer cell resistance to glucose starvation and cell death by anoikis^42^, the mechanistic details of which remain to be elucidated. It is unclear that the PKA effects are through ABCB1 upregulation as some groups have shown that PKA (and PKC) are essential for the translocation and function of ABCB1, whereas others have challenged these findings (reviewed in Ref.^43^). It may be that ABCB1 regulation by PKA is context-dependent and/or that the drug-resistant effects uncovered in this study upon *PRKACG* overexpression in Hap1 cells is ABCB1-independent. Interestingly, *HOXA5* and *HOXA6*, two genes involved in regulating human embryonic development and stem cell differentiation were also identified in our study as potential rocaglate-response modifiers. Both have previously been shown to affect cell survival. Knockdown of HOXA5 by siRNAs has been shown to overcome cytarabine resistance in leukemia cells^44^. Overexpression of HOXA6 has been shown to stimulate BCL-2 expression, thus promoting cell survival^45^. As far as we are aware, neither HOXA5 nor HOXA6 have been implicated in the regulation of *ABCB1*. However, another family member, HOXC6, has been found to activate the *ABCB1* promoter, and its suppression in the paclitaxel-resistant squamous cell carcinoma line, FaDu/PTX, leads to a reversal of drug resistance^46^. One possibility is that since the minor groove and major groove interactions of homeodomain-DNA interactions are quite similar^47^, overexpression of HOXA5 or HOXA6 may lead to cross-occupancy of the HOXC6 binding site (TAAT motif at -2243) within the *ABCB1* promoter, resulting in activation of *ABCB1* expression. Lastly, the finding that PAIP1 and PAIP2 can also affect rocaglate response is intriguing, although the effect observed is not nearly as pronounced as with FOXP3 or NR1I3 (Fig. 2c). Both PAIP1 and PAIP2 are regulators of protein synthesis with opposing activities—PAIP1 is a stimulator of translation, whereas PAIP2 is an inhibitor of translation^48,49^. How overexpression of PAIP1 and PAIP2 can lead to increased drug resistance and whether this is reliant on a translation-based mechanism remains to be addressed. Whether co-expression of combinations of the “resistance” genes will lead to additive or synergistic effects is a point that will require assessment.

Our results demonstrate that response to Pat A, another eIF4A inhibitor that also stabilizes eIF4A onto RNA, is affected by exogenous expression of *FOXP3* and *NR1I3* (Fig. 3b). A synthetic analogue of PatA, des-methyl, des-amino (DMDA) pateamine A has been shown to be insensitive to ABCB1-mediated drug efflux^50^, indicating that the *ABCB1*-response to Pat A has been engineered out of the DMDA-PatA molecule. Ectopic expression of *FOXP3* and *NR1I3* did not confer cross-resistance to hippuristanol, another eIF4A inhibitor that functions by preventing RNA binding rather than by inducing clamping (Fig. 3b). These results are consistent with previous experiments assessing the IC_50_ of hippuristanol for viability in parental and Pgp-overexpressing HeLa cells, and which indicated that hippuristanol is not a substrate of ABCB1^29^.

Substituents at the C6 and C2 position of rocaglates are important determinants for Pgp efflux^51^ (Fig. 1a). Amide, methyl ester, and carboxylic acid substituents at C2, and the dioxyanyloxy side chain of silvestrol at C6 render rocaglates susceptible to drug efflux^51^. Rocaglates lacking these modifications while maintaining potency are less affected by ABCB1^52,53^ indicating that systematic structure–activity relationship studies can produce compounds that are recalcitrant to this mechanism of resistance.

Taken together, although it was previously determined that ABCB1 is a modifier of rocaglate-responsiveness^17^, our data extend these results and define regulatory factors upstream of *ABCB1* whose expression/activity can promote the emergence of drug resistance. Since rocaglates are being developed as anti-cancer therapeutics, this information is critical in determining a gene expression signature, and hence cancer type(s), against which these compounds would be most effective.

## Methods

### Compounds

Silvestrol, CR-1-31-B, and CMLD12612 used in this study were synthesized at Boston University as previously described^23,54,55^. The isolation of PatA from the marine sponge Mycale sp. has been previously described^56^. Hippuristanol was chemical synthesized^57,58^. Verapamil was purchased from Cayman Chemicals and elacridar and zosuquidar were from AdooQ^®^ Bioscience. Compound stocks were prepared in 100% DMSO, aliquoted, and stored at − 80 °C.

### Cell culture and lentiviral transduction

Hap1 and eHAP1 cells were obtained from Horizon Discovery and maintained in Iscove’s Modified Dulbecco’s Medium supplemented with 10% fetal bovine serum, 1% penicillin–streptomycin antibiotics (Pen-Strep), and 2 mM l-glutamine at 37 °C and 5% CO_2_. 293 T/17 cells were grown in Dulbecco’s Modified Eagle Medium supplemented with 10% bovine growth serum, 1% Pen-Strep, and 2 mM l-glutamine. Tests for mycoplasma contamination were routinely performed.

Lentiviral transduction was used to infect Hap1 cells in the genome-scale ORF screen as well as with individual clones. Virus production for single clones was achieved by transfecting 15 µg of a DNA mixture containing the ORF plasmid, psPAX2 (second-generation lentiviral packaging plasmid), and pCMV-VSV-G (envelope proteins) at a 4:2:1 ratio into 293 T cells using polyethylenimine^59^. Viral supernatant was then collected 48 h later and used to infect Hap1 cells at an MOI (multiplicity of infection) ≤ 0.3 in the presence of 8 µg/mL polybrene and by spinoculation. Thirty-six hours later, transduced cells were selected with 2 µg/mL puromycin for 2–3 days.

The CRISPR-modified eIF4A2- and eIF4A1^F163^^L^eIF4A2–eHAP1 cells have been previously described^21^.

### Genome-scale ORF screen

The MISSION^®^ TRC3 LentiORF collection (Sigma) provided by the Genetic Perturbation Service (GPS) of the Goodman Cancer Research Center and McGill Biochemistry Dept, was pooled and transfected into 293 T cells to produce the lentiviruses, as previously described^60^. Hap1 cells (10^8^) were then infected with the pooled viral supernatant with 8 µg/mL polybrene but omitting the spinoculation step. Medium was refreshed 10 h later and spiked with 2 µg/mL puromycin another 24 h later. Following selection for 2–3 days, cells were expanded and frozen.

The positive selection screen was then performed using 1.5 × 10^7^ cells under 5 nM CR-1-31-B selection. Following treatment for 14 days, genomic DNA was isolated using the Roche High Pure PCR Template Preparation Kit followed by an RNase A treatment. One microgram of DNA was then used in 48 2-step PCR reactions with barcoded Illumina sequencing primers and then with P5/P7 primers. The reactions were then purified using the Roche PCR Purification Kit. Samples were then sequenced at The Center for Applied Genomics at Toronto SickKids hospital on the Illumina HiSeq 2500 platform. The 50-base kit with 62 cycles and single-end reads was used to obtain the exact read-length needed for the library vector. Sequences were then deconvoluted, and candidate genes ranked for enrichment using the MAGeCK software^61^ (https://sourceforge.net/p/mageck/wiki/Home/).

### Plasmids

Individual ORF vectors from the MISSION^®^ TRC3 library were provided by the McGill Platform for Cellular Perturbation:

- GFP (BRDN0000559466), TSSK2 (TRCN0000467628), SIK1 (TRCN0000489745), HIST1H3F (TRCN0000468120), FOXP3 (TRCN0000468197), RPSA (TRCN0000480498), SYNPR (TRCN0000473588), PRKACG (TRCN0000487900), ATP6V1C2 (TRCN0000466525), HOXA5 (TRCN0000466176), NR1I3 (TRCN0000487860), HOXA6 (TRCN0000480809), BCL2L1 (TRCN0000489920), PAIP2 (TRCN0000478712), PAIP1 (TRCN0000472903), and ABCB1 (TRCN0000488873).

### Compound titration and short-term growth assays

Hap1 cells were seeded in 96-well plates (5000 cells/well) a day before adding compound at various concentrations and treated for 48 h. Cells were then washed with phosphate-buffered saline (PBS) and fixed with 100 µL 50% trichloroacetic acid (TCA) for a minimum of 30 min. Fixed cells were then washed with water, then stained with 100 µL Sulforhodamine B (SRB: 0.5 g/500 mL H_2_O) for a minimum of 1 h. A gentle wash with 1% acetic acid then followed and plates were dried. The stain was re-suspended in 100 µL Tris–HCl buffer (pH 9), and read on a SpectraMax M5 plate reader for absorbance at 550 nM (OD_550_). Background absorbance values (empty wells) were then subtracted from each sample’s value, and the resulting number was normalized to the value obtained in the presence of DMSO.

### Competition assays

GFP-expressing Hap1 cells (2 × 10^4^) were mixed with 2 × 10^4^ candidate gene-expressing cells and read on a Guava EasyCyte HT Flow Cytometer (Millipore), then seeded in the well of a 24-well plate. Treatment was then started 24 h later, and cells were passaged and read on the flow cytometer every 3 days. Upon re-seeding, compound was re-applied 10 h later on the same day.

### Long-term growth assay

Colony formation assays were performed by seeding 2–4 × 10^4^ cells into 6-well plates. Time of fixation was chosen at the time when a single condition/well became confluent within an experiment. The time varied due to difference in resistance between FOXP3-, NR1I3-, and PRKACG-expressing cells, but ranged from 4 to 21 days. For DMSO, that was usually day 6. Cells were fixed with 4% formalin and stained with 0.1% w/v crystal violet before being photographed.

### Doubling time determination

Cells (5 × 10^4^) were seeded in 24-well plates in triplicate and counted every 2 days using a Bio-Rad TC20 automated cell counter and plotted. Dilution factors were accounted for, and GraphPad Prism 8 was used to transform the counts into their natural logarithms. The ‘exponential growth with log(population)’ non-linear fit was then applied, allowing for determination of doubling time.

### RNASeq

Hap1 cells (5 × 10^6^) were seeded in 10 cm dishes and allowed to expand for 48 h. Cells were then washed with PBS and RNA was harvested using the Qiagen RNeasy Mini Kit with on-column DNase I digestion. Samples were then sent to the Institut de Recherches Cliniques de Montréal (IRCM) for library preparation and deep sequencing. RNA integrity was first assessed on a Bioanalyzer RNA pico chip (Agilent), then strand-specific, barcoded libraries were prepared following ribosomal RNA (rRNA) depletion. Deep sequencing was performed using the Illumina HiSeq 4000 platform with paired-end 50 reads at approximately 50 million reads per sample. Biological triplicates for both *GFP-* and *FOXP3*-expressing cells were obtained.

Sequencing reads were then analyzed for quality using FastQC (available at: http://www.bioinformatics.babraham.ac.uk/projects/fastqc), and adapters were removed using Trimmomatic^62^. Read mapping to the UCSC hg38 genome assembly was then performed using TopHat^63^, in conjunction with Bowtie^64^. Reads mapping onto gene exons were counted using featureCounts^65^ and quantile normalized in each sample using preprocessCore^66^. Differential gene expression analysis was then performed using edgeR^67^.

### GO analysis

Gene ontology analyses were performed by uploading the significantly differentially expressed genes to the online Database for Annotation, Visualization and Integrated Discovery (DAVID) v6.8 tool^68^.

### RNA isolation and RT-qPCR

RNA was isolated using TRIZOL (Invitrogen) as per the manufacturer’s instructions. RNA (1 µg) was used for cDNA synthesis with M-MuLV Reverse Transcriptase (New England Biolabs, NEB). cDNA was then diluted 1:10 and 1 µL was used in a 10 µL qPCR reaction as outlined in the SsoFast EvaGreen Supermix (Bio-Rad) protocol. The 2^−ΔΔCT^ method was then used to calculate relative enrichment of transcripts.

Primers were designed using PrimerBank^69–71^ and obtained from BioCorp. For *ABCB1* (ABCB1_forward, ^5′^TTGCTGCTTACATTCAGGTTTCA^3′^; ABCB1_reverse, ^5′^AGCCTATCTCCTGTCGCATTA^3′^) and *GAPDH* (GAPDH_forward, ^5′^GGAGCGAGATCCCTCCAAAAT^3′^ and GAPDH_reverse, ^5′^GGCTGTTGTCATACTTCTCATGG^3′^).

## Supplementary Information

Supplementary Figures.

Supplementary Table S1.

Supplementary Table S2.

## Data Availability

RNASeq data are available in the National Center for Biotechnology Information GEO database under the following accession number: GSE167242.
