# Supplementary material for: A forward genetic screen identifies modifiers of rocaglate responsiveness
Source: Sci Rep. 2021 Sep 16;11:18516. doi: 10.1038/s41598-021-97765-8 (PMC8445955; doi:10.1038/s41598-021-97765-8)
Supplement: Supplementary file 1 — Supplementary Figures. [file 41598_2021_97765_MOESM1_ESM.pdf]

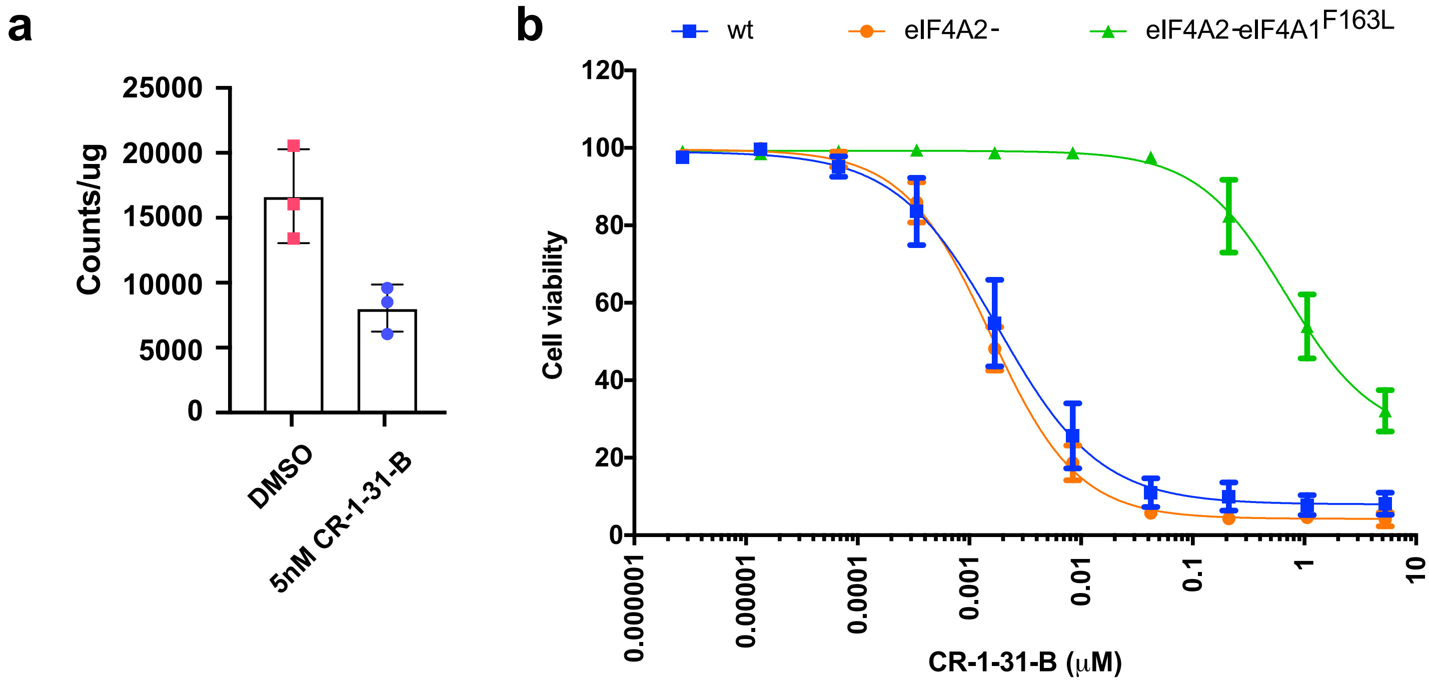

Figure S1. Biological effects of 5 nM CR-1-31-B towards Hap1 cells. **a.** Inhibition of protein synthesis in Hap1 cells following exposure to 5 nM CR-1-31-B for 48 h. One hour before termination of the experiment, cells were washed and incubated with Met-free media supplemented with 10% dialyzed serum. Fifteen minutes before the end of the experiment, ^{35}S-Met/Cys labelling mix (30 μCi) was added. Following labelling, the amount of radioactive protein was determined by TCA precipitation and standardized to total protein levels. n=3, p = 0.2. **b.** Cytotoxicity of CR-1-31-B towards wt (blue squares), EIF4A2- (orange circles), and EIF4A1^{F163L}EIF4A2- (green triangles) eHAP1 cells. Cells were exposed to the indicated concentrations for 48 h and viability was measured using the SRB assay. The IC50's of CR-1-31-B towards the test cell lines were: CR-1-31-B/eHAP, 2 ± 0.4 nM; CR-1-31-B/EIF4A2-, 1.4 ± .2 nM; CR-1-31-B /EIF4A1F163LEIF4A2-, 0.65 ± 0.2 μM; n = 4 ± SEM.

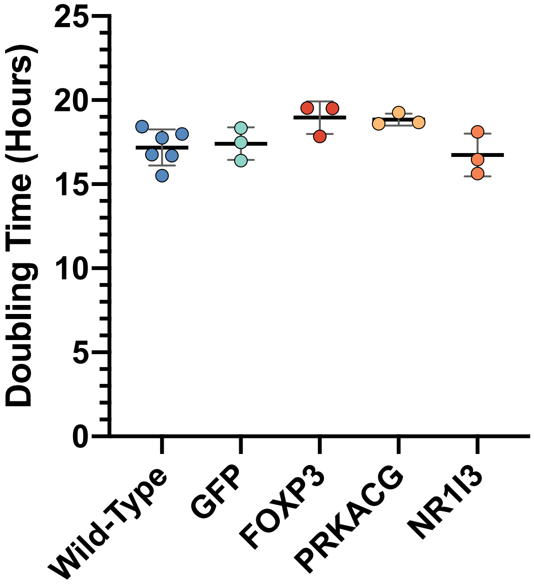

Figure S2. Doubling time of the indicated wt and transduced Hap1 cells. Doubling time was assessed as detailed in the Materials and Methods. n = 3 ± SD. No signficant difference between doubling times ($p > 0.05$) for all comparisons to wild-type Hap1 cells.

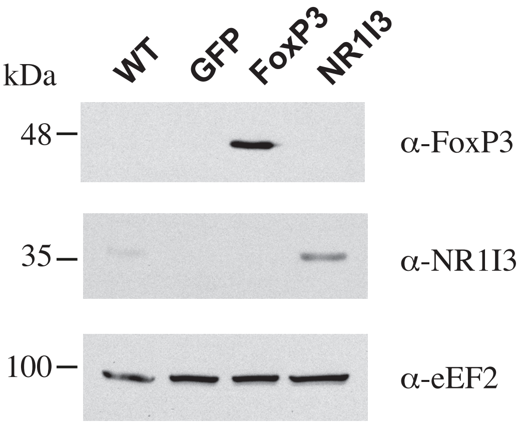

Figure S3. Western blot documenting expression of endogenous FOXP3 and NR1I3 proteins in wt Hap1, GFP-transduced, FoxP3-transduced, and NR1I3-transduced cells. Uncropped scans are in Fig S6.

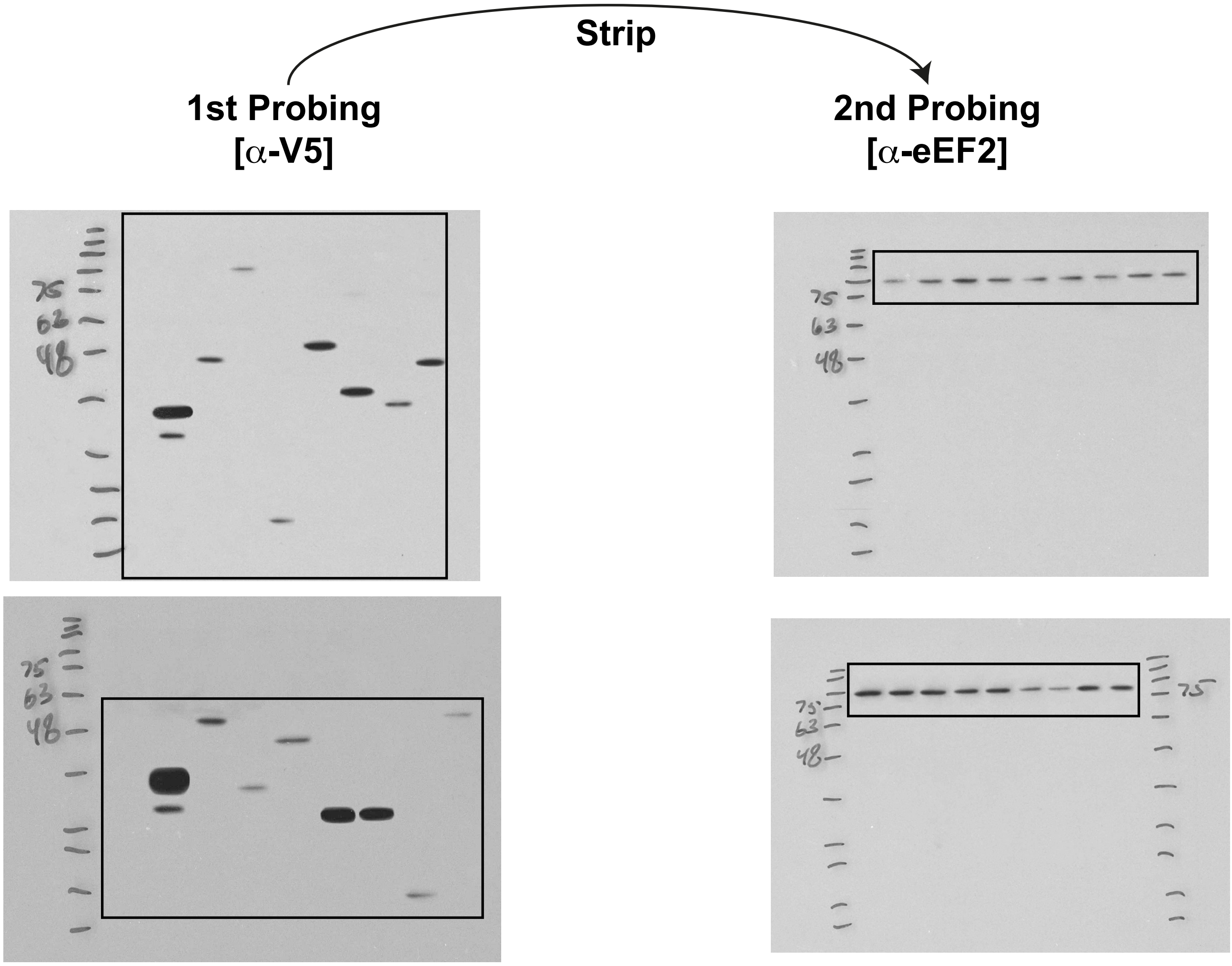

Figure S4. Uncropped blots for Fig 2a showing first and second probings.

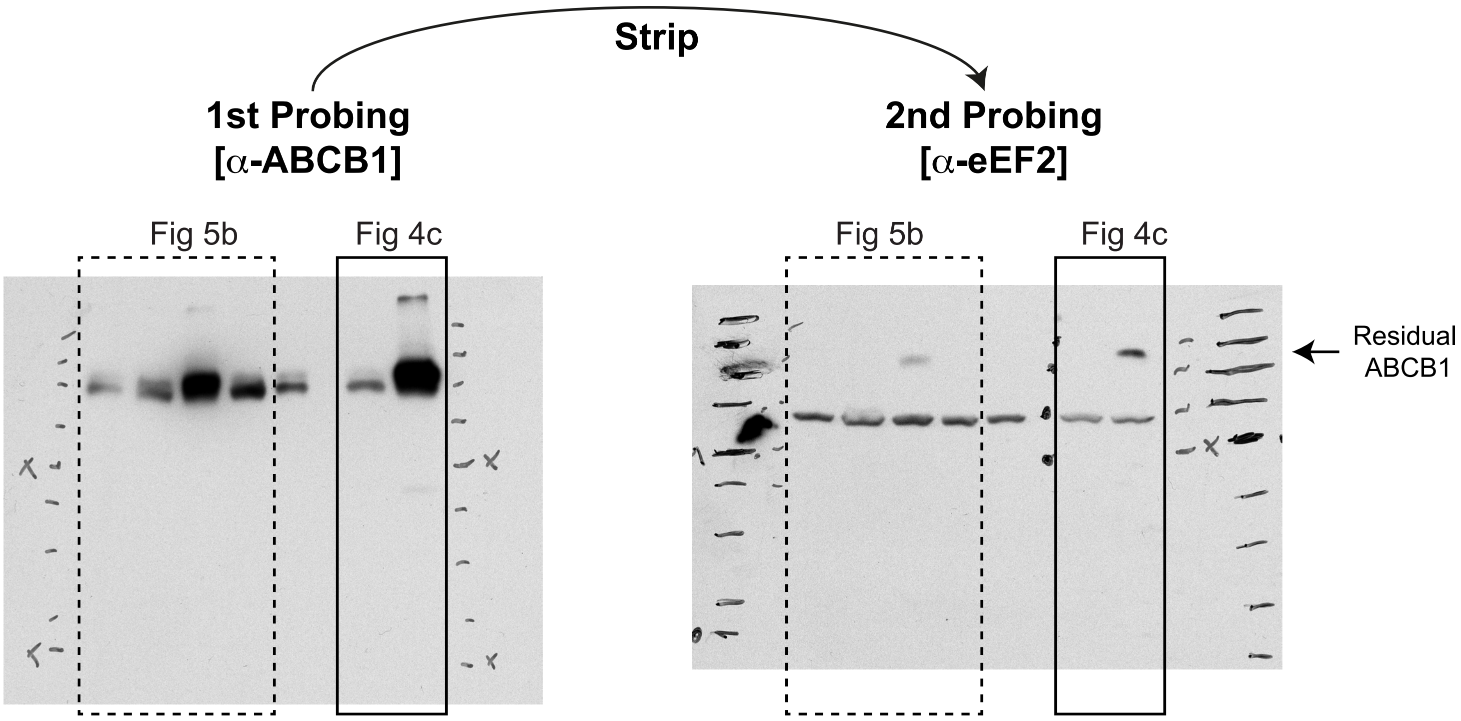

Figure S5. Uncropped blots for Fig 4c and 5b showing first and second probings.

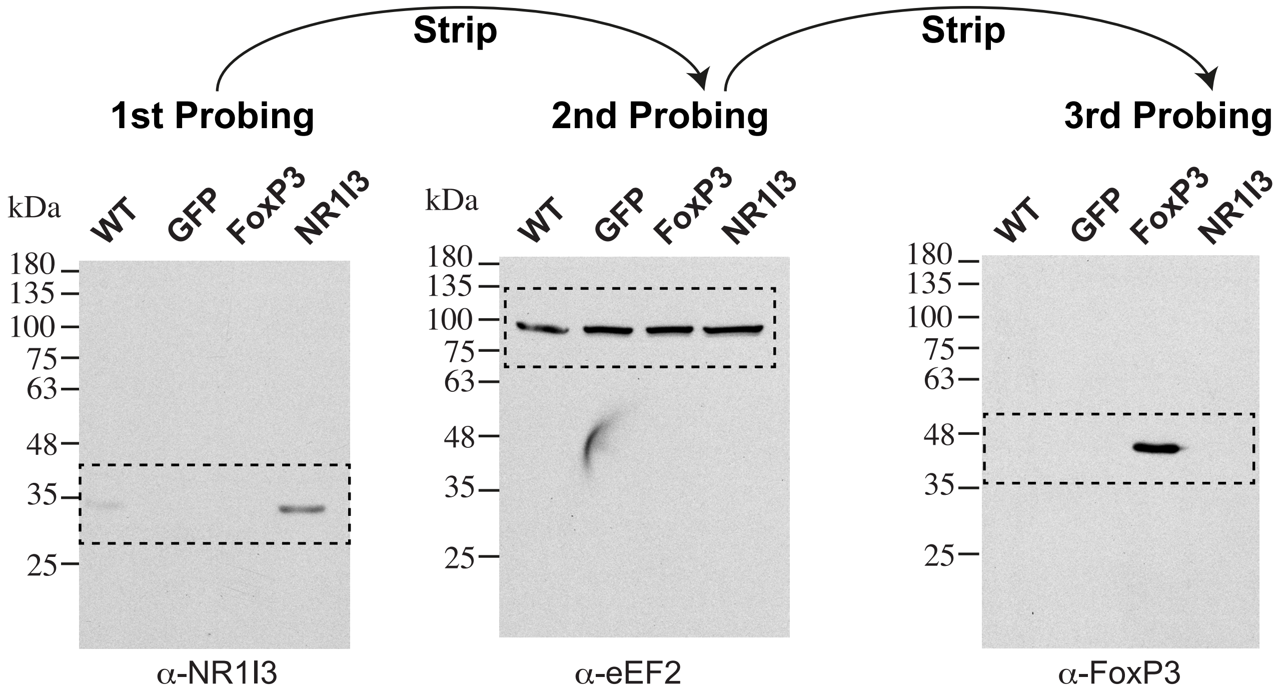

Figure S6. Uncropped blots for Fig S3 showing different probings.
